# Training Australian general practitioners to counsel women experiencing intimate partner abuse (WEAVE): a pre-post training analysis

**DOI:** 10.1186/s12875-024-02337-0

**Published:** 2024-03-20

**Authors:** Felicity Young, Mohajer Hameed, Leesa Hooker, Angela Taft, Kelsey Hegarty

**Affiliations:** 1 La Trobe Rural Health School, La Trobe University, Bendigo, VIC 3552 Australia; 2 Judith Lumley Centre, School of Nursing and Midwifery, La Trobe University, Bundoora, VIC 3086 Australia; 3 The Bouverie Centre, La Trobe University, Brunswick, VIC 3056 Australia; 4 Department of General Practice, The University of Melbourne, 200 Berkeley Street, Carlton, Vic 3053 Australia; 5 The Royal Women’s Hospital, 20 Flemington Road, Parkville, Vic 3052 Australia

**Keywords:** Domestic violence, Intimate partner abuse, General practice, Primary care, Training, Healthcare

## Abstract

**Background:**

Evaluations of Intimate Partner Abuse training for general practitioners is limited. The Women’s Evaluation of Abuse and Violence Care study trialled in Australia was a primary care intervention that included delivering the Health Relationships training, a program that educates practitioners on how to provide supportive counselling and assistance to women afraid of an intimate partner. We report on effectiveness of the Healthy Relationships training program within a cluster-randomised controlled trial.

**Methods:**

General practitioners filled out a baseline survey and surveys before and after training, including quantitative and open-text questions on barriers and enablers to supporting victim-survivors. The Physician Readiness to Manage Intimate Partner Violence Survey (PREMIS) tool, a validated measure, was included to assess practitioner knowledge, skills, confidence, and attitudes. General linear model repeated analysis of variance tested the difference between trial groups over time.

**Results:**

Fifty-two general practitioners completed the baseline demographic survey, with 65% (19 intervention, 18 comparison) completing both pre-and-post-training surveys. There were no between-group differences in baseline characteristics. Post-training, the intervention group had significantly higher average scores than the comparison on perceived preparation to address abuse (*p* = .000), perceived knowledge (*p* = .000), actual knowledge (*p* = .03), and greater awareness of practice-related issues (*p* = .000). There were no between-group differences in PREMIS opinion domain scores on workplace issues, self-efficacy and understanding of victims. Post-training, the qualitative data indicated that the intervention practitioners (*n* = 24) reported increased knowledge, awareness, and confidence, while time pressures and lack of referral options impeded addressing abuse.

**Conclusion:**

The Healthy Relationships Training program for general practitioners increased aspects of practitioner knowledge, skills, and confidence. However, more support is needed to change opinions and support victim-survivors sustainably.

**Trial registration:**

The WEAVE trial was registered on 21/01/2008 with the Australian New Zealand Clinical Trial Registry, number ACTRN12608000032358.

**Supplementary Information:**

The online version contains supplementary material available at 10.1186/s12875-024-02337-0.

## Background

Intimate partner abuse (IPA) is a global public health issue that disproportionately affects women and girls, violating their human rights and overall health and well-being [1]. The World Health Organization, London School of Hygiene & Tropical Medicine and South African Medical Research Council [2] define IPA as any behaviour or action perpetrated by a current or former partner that causes physical, psychological/emotional, sexual, or economic harm. The most common form of IPA is within heterosexual relationships, with men using violence against women and their children [2]. While gender is a common element of IPA, some groups’ experiences are compounded by various intersecting factors, such as disability, indigeneity, gender identity, immigration status, and poverty [2]. Victim-survivors of IPA experience poor physical, mental, sexual, and reproductive health, increasing their utilisation of healthcare services [2].

### IPA and Healthcare

Because IPA is a significant health issue, victim-survivors experience poor health compared to non-affected persons, including physical injuries, chronic pain, mental health problems (post-traumatic stress disorder, depression, anxiety) and reproductive health issues [2, 3]. Healthcare services that provide care to women and their children, particularly primary care services, are ideally positioned to identify and respond to IPA [4]. Although evidence from high-income countries indicates that women who experience IPA frequently attend primary care services [2], few are identified by general practitioners (GPs) [5]. As victim-survivors most often disclose to GPs, it is essential that they are equipped to support and improve outcomes for patients experiencing IPA [6].

Healthcare has a crucial role as the first point of contact for many victim-survivors [1]. The World Health Organization, London School of Hygiene & Tropical Medicine and South African Medical Research Council [2] recommend that health providers routinely assess high-risk groups for IPA, be trained in IPA risk assessment and be able to provide supportive counselling [1, 7]. A Cochrane systematic review of 19 trials of health practitioner training for IPA, mostly from the USA, found that the strength of evidence was low to very low [8]. There was some evidence that training health practitioners to respond to IPA may impact practitioners’ self-reported attitudes, knowledge, and readiness to respond to IPA. However, training interventions may make little to no difference on practitioner’s referral practice and there are uncertain effects on well-being and health outcomes of victim-survivors [8]. The authors of the review call for further research to assess impacts of IPA training.

### WEAVE intervention

The Women’s Evaluation of Abuse and Violence Care (WEAVE) study was the first in Australia to determine if a multifaceted intervention in primary care could increase the quality of life, mental health and safety behaviours for women experiencing IPA [9]. The WEAVE [10], cluster randomised control trial (RCT), provided a professional intervention (WEAVE Healthy Relationships Training) to GPs in Victoria, Australia, on how to respond to IPA when women experiencing abuse were identified, and to support them with brief counselling sessions [9]. The study addressed the evidence gap in how to improve outcomes for women experiencing IPA through a structured intervention [7].

The WEAVE intervention utilised a combination of methods to develop the Healthy Relationships Training and brief counselling sessions [10]. The counselling sessions were designed based on the Psychosocial Readiness Model [11–13], systematic reviews of healthcare-based interventions [14], meta-analysis [15] of qualitative studies and international consensus IPA primary care guidelines [16, 17]. The Healthy Relationships training developed from this evidence base has laid the foundation for IPA guidelines and training for GPs in Victoria and Australia [18], as well as being adapted as part of other Australian IPA primary care interventions [19]. The overall trial findings reported elsewhere [7] showed that the intervention did not change women’s quality of life. Nevertheless, women’s depression symptoms improved and this was mediated by the level of GP inquiry and GP support [7]. However, the impact of the WEAVE training on GPs’ self-reported IPA knowledge, confidence, attitudes, and skills in identifying and responding to women are yet to be explored. Therefore, this analysis of the Healthy Relationships training program aims to highlight the impact of this program on GP knowledge of IPA and their ability to improve outcomes for women experiencing IPA.

## Methods

### Design and participants

This paper outlines the evaluation of the Healthy Relationships Training program included in the WEAVE project. WEAVE, a cluster RCT [9], recruited Victorian GPs and their female patients (aged 16–50 years) who screened positive for being afraid of their partner or ex-partner. GPs from both urban and rural areas were recruited between January 2008-January 2010 and randomised between September 2008-June 2010. Eligibility included working three or more sessions per week; used electronic records and that 70% or more of their patients spoke English (based on GP’s self-report and medical records). GPs and their patients were randomly allocated to intervention and comparison groups once all baseline data had been collected. The WEAVE intervention method is described elsewhere [10], but in brief it included researchers inviting and training GPs to participate in the RCT, researchers inviting female patients via mail to complete a brief survey, screening them for a positive result and then notifying GPs when a female patient screened that they were afraid of their partner and then inviting them for brief GP counselling for relationship and emotional health issues [10].

The GPs in the intervention group received the Healthy Relationships Training program that aimed to train practitioners to respond to victim-survivors of IPA and deliver a brief counselling intervention, encourage safety planning and referral. The training included four hours of self-directed learning, two hours of teleconferences and two one-hour in-practice sessions delivered by a GP academic facilitator with a simulated patient [9]. Post recruitment, the program [10] was completed with the intervention group over six weeks during December 2008-January 2011 and included:

- An audit of 20 consecutive patients followed by a teleconference to reflect on the audit and GPs experiences of managing IPA (2 h).
- Distance education program including guidelines, demonstration videos of survivors’ voices and a health practitioner consultation (2 h).
- Two practice visits by a GP facilitator 1–2 weeks apart (3 h). The first session focused on active listening, attitudinal change and managing safety and confidentiality. The second session used a simulated patient, role playing different readiness for action scenarios (including a fishbowl activity). This session focused on motivational interviewing and problem-solving techniques and when to use these different approaches. The brief intervention included discussing the option of referral and collaborative patient care with other health and social services. Examples of practical exercises and role-playing guidelines are published elsewhere [10].
- Follow up teleconference to discuss any barriers or enablers to GPs putting what they learned into practice (1 h).
- Use of survivors’ voices and modelling of non-abusive behaviors in training interactions with health professionals as part of the hidden curriculum [20].

GPs in the comparison group received a brief education pack that included fact sheets and a journal article on managing IPA.

The PREMIS (Physician Readiness to Manage Intimate Partner Violence Survey) tool [21] was used as a reliable and comprehensive measure of GPs’ IPA knowledge, attitudes, and practices to assess training effectiveness. The specific PREMIS domains used in this study included perceptions of partner abuse (perceived preparation, perceived knowledge), actual knowledge, opinions (preparation, workplace issues, self-efficacy, alcohol/drugs, and victim understanding), and practical issues. See Additional file 1 for more information on the PREMIS Tool.

### Sample

Fifty-two GPs were randomised into intervention (25) and comparison (27) groups, with stratification by GP practice location, with a random permuted block size of two and four within each stratum [7]. Informed consent was obtained by all GPs before completing the PREMIS tool.

### Quantitative analysis

Descriptive statistics were used to summarise GP’s characteristics and outcomes pre-and post-training. Means and standard deviations were used for continuous variables, and frequencies for categorical variables. Repeated measures MANOVA, with time as the within-subjects variable (pre-post training measurements) was used to explore differences in training outcomes between GPs who received the training (intervention group) and GPs who did not receive any training (comparison group) [22].

### Qualitative analysis

Open-text responses from intervention group participants at baseline, pre-and-post-intervention surveys were analysed using an inductive content analysis approach [23]. The responses were reviewed by the lead author, with initial codes or keywords identified from each response. Keywords were then thematically grouped based on similarity or relevance. Quotes that reflected and supported the themes were extracted for inclusion in this paper and reviewed by the senior author.

The trial conformed to the CONSORT guidelines [24]. Ethical approval was provided by the University of Melbourne Human Research Ethics Committee.

## Results

### GP sample

Fifty-two GPs completed the baseline survey including sociodemographic information (see Table 1). A majority of 61.5% of GPs identified as female, with 71.2% located in urban Victoria with a mean age of 48.1. Over 80% of GP had completed their medical education in Australia. Previous IPA training varied, with 53.3% having completed two or less hours of training with only 15.6% having completed over six hours of IPA training. Less than half (42.9%) of the GPs had undertaken mental health training.

**Table 1:** Characteristics of participants (general practitioners) in the WEAVE trial and training

		Group Characteristics Frequencies (%)		Group differences	
	Total (*n* = 52)	Comparison (*n* = 27)	Intervention (*n* = 25)	(*p*. value)	Australia ^(a)^
Urban ^(**b)**^	**37** (71.2)	**19** (70.4)	**18** (72.0)	0.897	89.3
Female	**32** (61.5)	**18** (66.7)	**14** (56.0)	0.430	39.4
Age, yrs. M (SD)	**48.1** (8.1)	**46.9** (7.7)	**49.3** (8.4)	0.312	49.3
Graduated in Australia (yes)	**37** (80.4)	**18** (78.3)	**19** (82.6)	0.138	74.3 ^(d)^
Time since graduation, yrs. M (SD)	**23.5** (8.4)	**22.3** (8.3)	**24.6** (8.6)	0.371	
GP experience, yrs. M (SD)	**17.6** (7.9)	**16.8** (7.3)	**18.4** (8.5)	0.133	
Works in group practice	**50** (96.2)	**27** (100.0)	**23** (92.0)	0.134	87.9
Clinical practice, hrs per week M (SD)	**33.6** (12.1)	**30** (12.1)	**36.6** (11.6)	0.088	38.3
Mental health training (yes)	**15** (42.9)	**7** (38.9)	**8** (47.1)	0.482	
IPA education, hrs					
<1	**19** (42.2)	**8** (36.4)	**11** (47.8)		
1–2	**5** (11.1)	**4** (18.2)	**1** (4.4)		
3–5	**14** (31.1)	**8** (36.4)	**6** (26.1)		
>6	**7** (15.6)	**2** (9.1)	**5** (21.8)	0.281	

Notes: IPA = Intimate Partner Abuse; (a) 2009 AIHW Medical Labour Force Survey; (b) RRMA classification 1–2; (d) BEACH 2009; data is replicated from WEAVE published trial [1]

### Survey

**Fig. 1:**
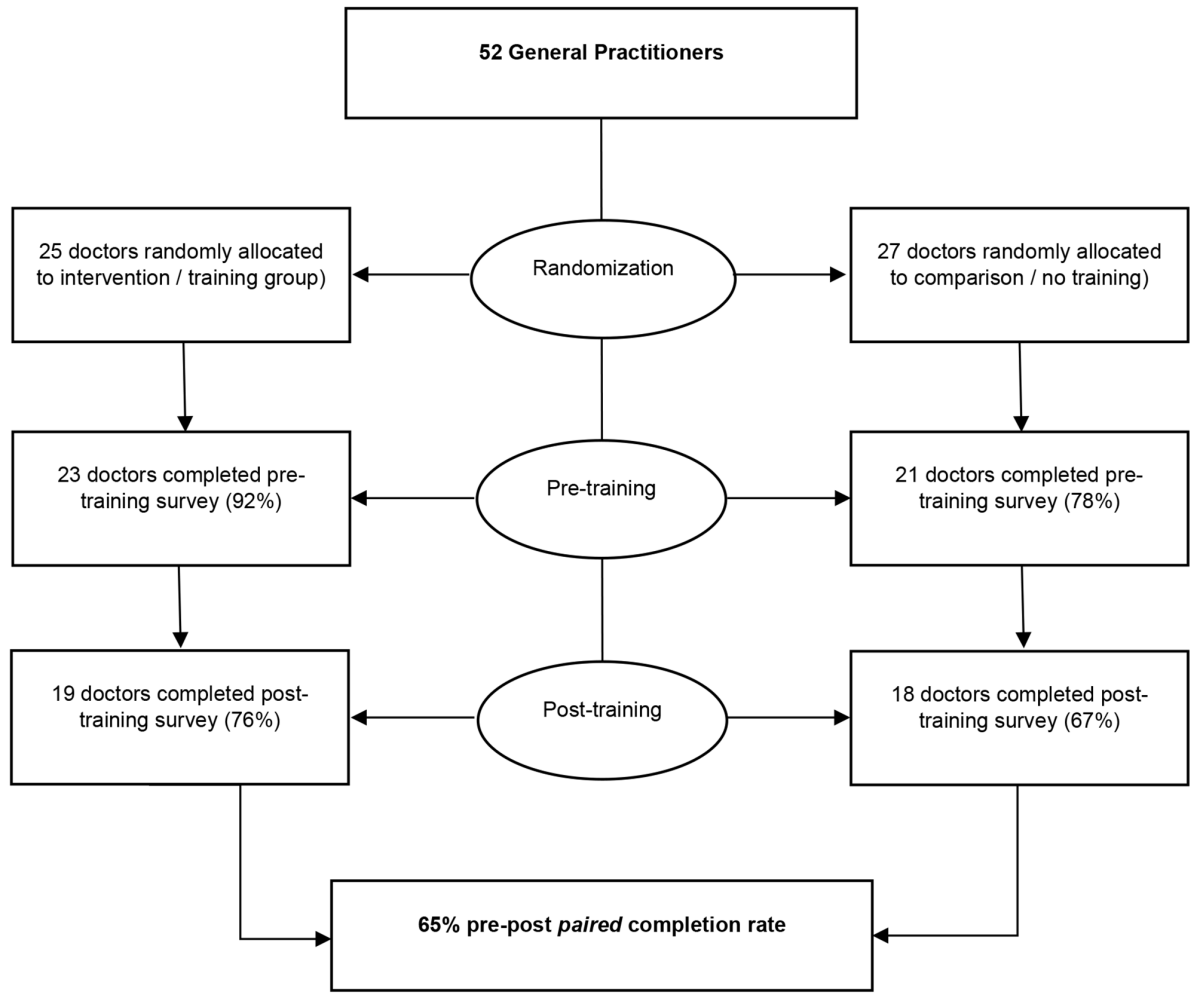
Participant flow chart and data completion rates

Whilst 52 GPs completed baseline survey, 65% (*n* = 34/52) completed all pre-and post-training surveys (see Fig. 1). There were no between-group differences in baseline characteristics (see Table 1). Whilst no statistically significant group differences were seen, there was a pattern that urban-based and female GPs were slightly over-represented in this sample [7].

Means and standard deviations for the PREMIS domains are presented in Table 2 for pre-and-post-training for both intervention and comparisons groups. Results indicate that post-training, the intervention group had higher mean scores on perceived and actual IPA knowledge(see Table 2). This means the intervention groups perceived knowledge, what they felt they knew about IPA identification and management and their actual knowledge about IPA identification and management, increased. See Additional file 1 for more information on perceived and actual knowledge measures. The repeated measures MANOVA, with time as the within-subjects variable, revealed a significant difference in perceived knowledge between intervention and comparison group (*F*(1,32) = 19.56, *p* < .001, *partial eta squared* = 0.379). In comparison to the control group, the intervention group showed higher average scores on perceived preparation to address IPA (*F* (1,30) = 16.01, *p* = .000, *partial n*^*2*^ = 0.348), perceived IPA knowledge (*F* (1,32) = 19.56, *p* = .000, *partial n*^*2*^ = 0.379), actual IPA knowledge (*F* (1,32) = 5.15, *p* = .030, *partial n*^*2*^ = 0.139), and greater awareness of practice issues (*F* (1,32) = 10.51, *p* = .003, *partial n*^*2*^ = 0.247). Results detected no between group difference in PREMIS opinion domain scores of preparation, workforce issues, self-efficacy, alcohol and other drugs, and victim understanding. Both intervention and comparison groups had similar pre-post training scores across these domains (see Table 2).

**Table 2:** Differences between study groups over time by PREMIS scales

	Group comparison Mean (Standard Deviation)				Group differences			
PREMIS Scales	Comparison (*n* = 17)		Intervention (*n* = 17)		Interaction effect	Effect Size	Main effect	Effect size
**Background**:	Pre	Post	Pre	Post				
• Perceived preparation	4.65 (1.21)	4.74 (1.22)	3.83 (1.11)	5.54 (0.68)	F (1,30) = 16.01, *p* = .000	0.348	F (1,30) = 20.06, *p* = .000	0.401
• Perceived knowledge	4.06 (1.23)	4.25 (1.37)	3.54 (1.03)	5.11 (0.67)	F (1,32) = 19.56, *p* = .000	0.379	F (1,32) = 31.85, *p* = .000	0.499
**Actual knowledge**								
• Actual knowledge	27.17 (3.03)	26.12 (7.41)	26.59 (2.72)	29.29 (3.24)	F (1,32) = 5.15, *p* = .030	0.139	F (1,32) = 0.986, *p* = .328	0.030
**Opinions**								
• Preparation	3.26 (0.94)	3.43 (0.72)	3.29 (0.67)	3.96 (0.89)	F (1,30) = 2.27, *p* = .142	0.070	F (1,30) = 6.57, *p* = .016	0.180
• Workplace issues	3.57 (0.54)	3.63 (0.61)	3.52 (0.45)	3.83 (0.53)	F (1,31) = 2.13, *p* = .154	0.065	F (1,31) = 4.63, *p* = .039	0.130
• Self-efficacy	3.02 (0.45)	3.11 (0.67)	2.73 (0.53)	2.91 (0.48)	F (1,32) = 0.28, *p* = .601	0.009	F (1,32) = 2.51, *p* = .123	0.073
• Alcohol/drugs	3.63 (0.58)	3.62 (0.73)	3.44 (0.57)	3.45 (0.57)	F (1,30) = 0.01, *p* = .958	0.000	F (1,30) = 0.01, *p* = .958	0.000
• Victim understanding	3.78 (0.67)	3.81 (0.58)	3.57 (0.38)	3.91 (0.54)	F (1,32) = 3.15, *p* = .085	0.090	F (1,32) = 4.46, *p* = .042	0.122
**Practice Issues**								
• Practice issues	10.14 (4.51)	8.65 (5.10)	7.36 (3.41)	11.77 (5.81)	F (1,32) = 10.51, *p* = .003	0.247	F (1,32) = 2.57, *p* = .118	0.074

Note: all scores treated as continuous data with higher scores indicating better outcome (some negatively worded items reversed)

### Open-text responses

Twenty-four (96%) GPs in the intervention group contributed to open-text responses in the three surveys. Four themes were identified post training from the intervention group: (1) increased knowledge and awareness of IPA, (2) GPs gained confidence in identifying and responding to IPA, (3) time pressures impacting on GPs perceived self-efficacy and (4) available referral pathways to support GPs and patients.

### Increased GPs’ knowledge and awareness of IPA

In the baseline and pre-training surveys, an overwhelming majority of respondents in the intervention group (91.7%) reported a lack of knowledge, skills, and awareness of IPA in their patient population.

‘This is an area I feel I do badly at and need further knowledge.’ – Male, Urban GPs (intervention group, pre-training survey #3).

‘[I want to] Improve knowledge re: specific psychological issues and evidence of what works when helping women and improved knowledge re: referral options.’ – Female, Urban GP (intervention group, pre-training survey #4).

Post-training surveys reported an increase in IPA knowledge and awareness which was noted in the intervention group responses. These respondents acknowledged that a patient’s readiness to change may vary, and ongoing support and validation of experience was essential.

‘[I am] more aware of the very long-term damage from being in an abusive relationship - some of the women I counselled had been out of the relationship for many years, but it was still having a very major impact on their lives.’ – Female, Urban GP (intervention group, post-training survey #4).

‘To realise how much I had failed to recognise the extent and nature of the problem and to correct the habit of doing so; to correct my tendency to attribute blame to the victim for apparent provocation and failure to leave’ – Male, Rural GP (intervention group, post-training survey #14).

### GPs gained confidence in identifying and responding to IPA

In the baseline and pre-training surveys, intervention GPs (n = 16) noted that they were not confident in asking about IPA and how to support patients if they disclosed IPA.

‘Not knowing who/ how to ask’ – Female, Rural GP (intervention group, baseline survey #21).

‘Lack of confidence in knowing what to do once they disclose.’ – Female, Rural GP (intervention group, pre-training survey #38).

‘…GP having the interest and time to spend with her and the knowledge to discuss safety issues after assessing her risk of being harmed by her partner’ – Female, Rural GP (intervention group, pre-training survey #38).

However, in post-training surveys collected after the training, intervention GPs self-reported an increase in confidence, a greater understanding of the impact of IPA on victim-survivors, and an improved ability to facilitate discussions about IPA and establish patient safety.

‘[I gained] more confidence in asking about IPA, increased awareness of the possibility of IPA, more understanding of the effects of IPA, more confidence in being able to assist women experiencing IPA.’ – Female, Rural GP (intervention group, post-training survey #38).

‘[I am now] comfortable asking sensitive questions. Will ask more. Assessing risk.’ – Female, Urban GP (intervention group, post-training survey #17).

### Time pressures with IPA patients impacted GP’s self-efficacy

In either the baseline or post-training survey, over half of the GPs (n = 16) in the intervention group mentioned time pressures limiting their ability to address IPA. Survivors often required lengthier consultations, longer than that for which GPs perceive they are poorly remunerated for, which impacted on their willingness to discuss IPA with patients.

‘Poor remuneration for prolonged consultations’ – Female, Urban GP (intervention group, baseline survey #37).

‘Time, time, time. I wish I can have more time talking to the patients to find out their real problems and to help them. – Female, Urban GP (intervention group, baseline survey #41).

‘Time may be a factor, but a longer consult can be arranged.’ – Male, Urban GP (intervention group, baseline survey #49).

Post-training survey’s respondents in the intervention group varied in their ability to manage the time pressures and the psychological impact of clinically supporting IPA patients, with some intervention GPs able to prioritise patients experiencing IPA, while others found it personally more challenging even after training.

‘I do find it stressful asking about the possibility of IPA as it takes time and sensitivity dealing with a positive response’ – Female, Rural GP (intervention group, post-training survey #38).

‘It takes more effort and is likely to put me more behind, so I do sometimes consciously make the decision not to ask about possible IPA, which I don’t feel good about’. – Female, Rural GP (intervention group, post-training survey #38).

### Available referral pathways supported GPs and their patients

In the post-training survey, intervention GPs (n = 21) reported that access to community resources and referral services enabled them to effectively support patients experiencing IPA.

‘Rapid access to affordable help and advice… Good knowledge of how to access other services.’ – Female, Urban GP (intervention group, post-training survey #4).

The training was able to increase intervention GPs’ knowledge of local resources available.

‘Increased understanding, knowledge, and skills and tools for assessing and supporting women with current or previous IPA.’ – Female, Rural GP, (intervention group, post-training survey #46)

‘[I have gained] greater knowledge of community resources.’ – Male, Urban GP (intervention group, post-training survey #5).

## Discussion

The WEAVE Healthy Relationship Training program delivered to GPs in Victoria has formed the foundation of IPA education for primary care services over the last decade and more recently has been rolled out nationally in Australia [18]. This analysis has identified that the WEAVE IPA training was successful in increasing the perceived and actual knowledge of GPs in the intervention group and is consistent with other studies on healthcare provider training to address IPA [8]. The intervention group’s greater awareness of practice issues in the post-training survey, highlights that GPs changed their actual practice with an increase in appropriate responses to IPA questions. This included: situations in which GPs ask about IPA, actions undertaken when IPA is disclosed/identified, IPA resources in the clinic and knowledge of IPA services in the community [21]. Increased knowledge and awareness of these practice issues is essential when managing IPA victim-survivors, and can address healthcare providers’ knowledge gaps, attitudes, and negative responses to IPA [8]. Given that victim-survivors of IPA expect healthcare practitioners to provide non-judgemental care and offer practical support, such as referrals [5], increasing GPs perceived and actual knowledge is critical.

Since the WEAVE training was completed, the Victorian State Government and Australian Federal Government actively engaged in rolling out IPA training to GPs [18, 25]. While there has been an increase in IPA awareness in the health sector, the issues highlighted in the qualitative analysis convey the GP and system level barriers and enablers to providing quality IPA care and support. Barriers to GPs include lack of time and perceived poor remuneration for engaging in IPA prevention and response. This limits GP self-efficacy and remuneration generally remains a controversial topic in current primary care policy. At a system level, a lack of ongoing training and education in identification and management of IPA patients also challenges the sustainability of the current gains in a complex GP clinic environment [6, 8]. This is a serious ongoing systematic issue. Without primary healthcare service reform, including IPA medical education and ongoing IPA professional development requirements, there is unlikely to be significant and sustainable change to GPs’ ability and willingness to identify and manage patients experiencing IPA. Wider systematic changes to the healthcare sector [1], in particular primary care, are needed [8] to enable health practitioners to strengthen their readiness to address IPA [6].

### Strengths and limitations

This paper provides analysis of the WEAVE trial’s training data, with the overall evaluation of the WEAVE trial completed in 2013 [7]. It provides the context for which most GP IPA training in Australia has been based. The training’s success in increasing perceived, actual knowledge and understanding of practice issues, which includes identifying more IPA patients and asking more frequently when indicators of IPA occur, highlights why this training has continued to be rolled out or adapted in the primary care setting. The research is limited by the available sample size of GPs who completed the pre-and post-surveys. However, the statistical significance of some of the PREMIS quantitative analysis and the supportive qualitative themes strengthen this research. The WEAVE training of GPs has contributed to further studies in which the training content is adapted to be culturally safe and inclusive care for migrant and refugee communities in the light of Australia’s diverse ethnic populations [19].

## Conclusion

It is essential for the wider healthcare sector and its funders, the Australian State and Federal Governments, to address the individual and systemic barriers impacting GPs ability to address IPA with the community. Without adequate time and perceived appropriate remuneration, and without increased knowledge and ongoing education in IPA identification and response, GPs will continue to struggle to safely and sustainability identify and support victim-survivors of IPA.

### Electronic supplementary material

Below is the link to the electronic supplementary material.

Supplementary Material 1

Supplementary Material 2

## Data Availability

The data that supports this study cannot be publicly shared due to ethical or privacy reasons and may be shared upon reasonable request to the corresponding author if appropriate.

## Declarations

This trial was registered on 21/01/2008 with the Australian New Zealand Clinical Trial Registry, number ACTRN12608000032358.

## Abbreviations

IPA: Intimate partner abuse
GP: General Practitioner
WEAVE: Women’s Evaluation of Abuse and Violence Care
PREMIS: The Physician Readiness to Manage Intimate Partner Violence Survey
RCT: Randomised control trial
