# Supplementary material for: Training Australian general practitioners to counsel women experiencing intimate partner abuse (WEAVE): a pre-post training analysis
Source: BMC Prim Care. 2024 Mar 20;25:93. doi: 10.1186/s12875-024-02337-0 (PMC10953085; doi:10.1186/s12875-024-02337-0)
Supplement: Supplementary file 1 — Supplementary Material 1 [file 12875_2024_2337_MOESM1_ESM.doc]

# Details

Name ­­___________________ Preferred Pseudonym _________________ Interview Date ­_________

# Before start tape

Plain language statement Consent form Audio-taping

# Introduction

I am interested in GPs experience of being involved in the weave project. Just to remind you **weave** aimed to evaluate if an intervention involving screening for intimate partner abuse by mailout, education and guidelines for GPs, and inviting women for a brief counselling intervention increased women’s safety, mental health and quality of life.

You were in the xx arm of the study and we are interviewing both women and GPs involved in both arms of the project.

Why- we hope to develop further interventions for GPs in this area, and help women to know how to get the most out of seeing their GP.

Anything you would like to ask about the project overall? How long have they got today – finish time? Note-taking, may check the tape.

# Opening Questions – the weave project

**Thinking back, what motivated you to join weave?**

**How did you find being involved in the project?**

**-**approach by research team

-time involved

-mailouts

-surveys

**Has it changed anything about the way you practice in this particular area? How?**

**If no**

**What could we have done to assist you in dealing with women experiencing family violence or intimate partner abuse?**

**How do you think your patients felt about being in the project?**

**Seeing abused women**

**How do you think an abused woman would feel as a patient in your practice?**

**–** in the waiting room, dealing with the receptionists

Do you know what the receptionists attitudes are towards abused patients?

Do you know anything about the attitudes of the other doctors in your practice?

**Can you tell me about a time when you saw an abused woman?**

- how did you know she was abused etc -if negative experience – what would have improved it

- do you think you met her needs

- how did you feel when you were seeing her

In general -is your gender important,

**Have your experiences changed over time?- How**

**Disclosure**

**How important is it for you to know whether your female patients’ are abused? When, why, relevance**

- have you ever asked in the last 12 months, what happened

- has anyone disclosed to you, what happened, how did you feel

- would you expect an abused women to tell you about abuse or not to tell, would you hope that she would tell you

- have you ever been surprised when told – what situations would be a surprise

- is her abuse relevant to her health care

**Responding and Training**

**What do you think are the most important things to do when you respond to a woman who is abused by her partner?**

**Have you had any training on this issue in the last few years? How did you find it? or**

*Intervention GPs* **Reflecting now on the weave training:**

What do you remember about the training?

How did you find the **weave** training?

-What was the main message about domestic violence that you took away from the training?

-Do you think that the training adequately prepared you to:

- - 1. Identify women at risk of domestic violence?

Yes □ No □

Comment?

- - 1. Talk to women about domestic violence?

Yes □ No □

Comment?

If not, what training or information would have been more useful?

-Are there any parts of the training that you have found to be particularly useful in your clinical practice? Could you elaborate a bit?

-Are there any other topics that you would have liked more information on during the training?

-Have you any comments or ideas about the formatting or length of training that might make the training more appealing to GPs?

**What features or changes would you like to see with training in this area?**

**Intervention (use intervention term or usual care depending on arm GP was in)**

What would you see as stopping women coming to GP counseling sessions for this issue?

What would you see as facilitators to women coming to GP counseling sessions?

What are your experiences of the **weave** counselling sessions or care you have offered to women who are abused in the last few years?

Would you recommend any changes to the intervention or care for abused women if it were to be offered to other people with similar issues?

-consultation

-systems

-resources

# Closing

**Is there anything you could change currently to improve your practice for abused women?**

- within the practice environment

- personally

**Is there anything else you would like to say/add?**

**How do you feel having talked about these issues?**

**Post interview**

**Contacts**

- Invite to contact me if would like to add something
- Can I contact him/her when transcribing to clarify an issue Yes No
- Would you like to read the transcript and have opportunity to comment and suggest changes Yes No

**Follow up**

- Would you like to participate in a follow-up interview or group discussion to hear about the early findings Yes No

**Abused women’s health resource**

**-** Would you like a copy of a resource I have developed containing information and referral resources? Yes No

Yes No

**Practice observation**

- would you be happy for me to observe the environment of your practice

(waiting room, brochures, reception area) and take a few notes about it? Yes No
