# Supplementary material for: Training Australian general practitioners to counsel women experiencing intimate partner abuse (WEAVE): a pre-post training analysis
Source: BMC Prim Care. 2024 Mar 20;25:93. doi: 10.1186/s12875-024-02337-0 (PMC10953085; doi:10.1186/s12875-024-02337-0)
Supplement: Supplementary file 2 — Supplementary Material 2 [file 12875_2024_2337_MOESM2_ESM.docx]

Additional file 1

PREMIS Tool

The PREMIS (Physician Readiness to Manage Intimate Partner Violence Survey) tool, is a survey that provides a comprehensive and reliable measure of clinician preparedness to manage patients experiencing intimate partner violence (IPV). The tool is publicly available and is used to measure the effectiveness of IPV education programs. It was adapted for the WEAVE intervention.

There are four key sections to the PREMIS tool:

1. Background – focused on the clinician’s previous type of IPV training, how much IPV training (in hours) they had received. Questions include identifying how prepared clinicians felt (perceived preparation) to manage IPV in a range of circumstances, measured from not prepared to quiet well prepared on a Likert 7-scale). It also includes questions (Likert 7-scale) to identify how much clinicians felt they knew about IPV (perceived knowledge) identification and management, from legal requirements to risk of IPV, the scale ranging from nothing (no knowledge) to very much (lots of knowledge).
2. Actual knowledge – focused on clinicians’ actual knowledge of IPV identification and management as an individual and within their clinic. This includes multiple questions about IPV patients attending their clinic, their role in referring IPV patients to other health and social services. It includes a section with statements that are required to be labelled true or false.
3. IPV Opinions – focused on clinicians’ attitudes and beliefs towards IPV. This section used a Likert 7-scale (strongly disagree to strongly agree) with multiple opinions stated related to IPV, with some purposefully worded negatively that were able to be reversed scored.
4. Practical issues – focused on self-reported behaviours as well as individual and clinic practices and policies for managing IPV patients. This includes multiple choice questions, questions about frequency of asking and care provided to patients when IPV indicators are presented and available resources and referral pathways at the clinic and the clinics processes.

Short LM, Alpert E, Harris JM, Surprenant ZJ: **A Tool for Measuring Physician Readiness to Manage Intimate Partner Violence**. *American Journal of Preventive Medicine* 2006, **30**(2):173-180.e119.

To access the PREMIS Tool-Kit instructions, which consists of the PREMIS instrument (includes the four key sections of the tool), codebook, and SPSS syntax and socring infomration please see: <https://www.futureswithoutviolence.org/userfiles/file/HealthCare/AJPM%20-Short+Toolkit.pdf>
